# Reprocessing of Simulated Industrial PLA Waste for Food Contact Applications

**DOI:** 10.3390/polym17182439

**Published:** 2025-09-09

**Authors:** Javiera Sepúlveda-Carter, Simón Faba, Marcos Sánchez Rodríguez, Marina P. Arrieta

**Affiliations:** 1 Departamento de Ingeniería Química Industrial y del Medio Ambiente, E.T.S. de Ingenieros Industriales, Universidad Politécnica de Madrid, C/José Gutiérrez Abascal 2, 28006 Madrid, Spain; simon.faba@alumnos.upm.es; 2 Grupo de Investigación: Polímeros, Caracterización y Aplicaciones (POLCA), 28006 Madrid, Spain; 3 The Circular Lab, ECOEMBALAJES España, C/del Cardenal Marcelo Spínola 14, 28016 Madrid, Spain; m.sanchezr@ecoembes.com

**Keywords:** reprocessed PLA, food contact materials (FCMs), non-intentionally added substances (NIASs), migration, sustainable packaging

## Abstract

This study explores reusing discarded industrial polylactic acid (PLA), such as defective parts, scraps and burrs, for food contact applications. Reprocessing of PLA (PLA-RP) was simulated via melt extrusion, and the obtained pellets were characterized in terms of viscosity average molecular weight (M_v_), melt flow index (MFI), the presence of non-intentionally added substances (NIASs) and the absence of metals to ensure that no substances exceeded the specific migration limits (SMLs). A slight reduction in the M_v_, accompanied by an increase in the MFI, was observed in PLA-RP. In virgin PLA, fewer compounds were detected, likely related to residual additives. A higher variety and concentration of volatile and non-listed compounds were observed in reprocessed PLA (PLA-RP), with three exceeding their assigned Cramer class thresholds, requiring further evaluation. Most identified substances were typically linked to thermal degradation or potential additives for reprocessing. In both the virgin and reprocessed materials, all substances with SMLs remained below applicable thresholds, including trace metals. The PLA-RP was further processed into films by means of a compression moulding process. The structure, mechanical behaviour, thermal stability and water vapor transmission rate were comparable to those of virgin PLA, indicating no significant changes. The overall migration level tested in a fatty food simulant remained below regulatory limits. The materials fully disintegrated under laboratory-scale composting conditions in less than 3 weeks. Thus, reprocessed PLA shows great potential as a non-migrating material of interest in the sustainable food packaging field.

## 1. Introduction

Due to the great impact on the environment of traditional plastics of fossil origin, the use of bioplastics has become a widely studied alternative due to their lower environmental impact. An example of this is polylactic acid (PLA), a biobased, recyclable and compostable biopolyester highly used in the food packaging sector [1,2]. In fact, PLA is currently processed at the industrial level with the same processing technology used for traditional petroleum-based thermoplastics (melt extrusion, injection moulding, thermoforming, foaming, electrospinning, 3D printing, etc.). As a food contact material, PLA is mainly commercialized for single-use disposal applications such as in bottles, cold drink cups, thermoformed and foamed trays, blister packages, overwrap and flexible films [2], but with somewhat limited applications due to its reduced thermal, mechanical and barrier properties [2,3,4]. Efforts are focused on increasing the useful life of this material [1,5,6,7,8,9,10], and at the same time, based on the objectives established for 2030 by the European Commission that seek to increase the content of recycled material in all plastic packaging, as an essential prerequisite to its strategy to introduce recycled plastics in a circular economy [11].

Considering food safety issues, the current legislation does not allow the direct use of recycled plastics coming from recycled streams for food contact applications due to the transfer of substances from packaging material to foodstuffs, which may affect food quality or even human health. Efforts are being made to improve end-of-life waste stream separation both technologically and at the consumer level [12]. Although PLA packaging is designed to be composted, it remains essential to ensure that such compostable materials do not contaminate traditional plastic recycling streams [13,14], in line with Regulation (EU) 2025/40 requirements [11].

In this context, during the industrial production of plastic products, several defective plastics parts and/or plastic burrs are discarded from the production line [1,8]. These discarded plastic pieces can be reprocessed and further used to produce reprocessed PLA pellets with a well-known origin and not coming from waste streams, which may requires additional washing steps, such as postconsumer PLA parts [10]. Regarding the reprocessed PLA’s properties, it has been observed that during successive reprocessing cycles, the main losses in melt flow index, viscosity average molecular weight and/or mechanical performance took place when PLA was subjected to more than four melt extrusion reprocessing cycles, while low PLA degradation was found between one and three reprocessing cycles [6,8,10].

On the other hand, in the case of food contact materials, it should be taken into account that some components might potentially migrate from the packaging material to the foodstuff, and currently complying with regulatory requirements presents additional challenges compared to the same virgin material [15]. In this context, additional sources of contamination are a major concern, with the presence of non-intentionally added substances (NIASs) being one of the main factors to consider [16].

The main goal of this research is to study the possibility to reintroduce PLA from the industrial trimming process (discarded defective PLA parts and/or plastics edges, burrs and scraps produced during manufacturing) into the food packaging sector. For this, the reprocessing of industrial PLA waste was simulated by reprocessing melt extruded PLA by a second melt extrusion step with the aim to simulate the industrial processing conditions of PLA products. As this PLA waste has already undergone thermal and mechanical stress, which may cause partial degradation (typically chain scission), the obtained reprocessed PLA was pelletized and further characterized in terms of the MFI and viscosity average molecular weight. In addition, the presence of volatile, semi-volatile and non-volatile substances, including potential NIASs, was evaluated to ensure food safety. Regarding inorganic substances, the presence of metals (Ba, Co, Mn, Zn, Cu, Fe, Li, Al, Ni, Eu, Gd, La, Tb, As, Cd, Cr, Pb, Hg, Sb) was also analyzed in 3% acetic acid (60 °C, 10 days), following the regulatory requirements for food contact materials. Films of both PLA and reprocessed PLA (PLA-RP) were produced by means of hot pressing, and their mechanical, structural, thermal, barrier, migration and disintegration properties under laboratory conditions were studied to obtain insights into the possibility to use this valorized plastic waste for film for food packaging.

## 2. Materials and Methods

### 2.1. Materials

Commercial-grade Ingeo^TM^ 2003D PLA with approximately 4.5% D isomer and with a density of 1.24 g·cm^−3^ was supplied by Natureworks (Minnetonka, MN, USA) [17].

### 2.2. Processing and Reprocessing of PLA

#### 2.2.1. PLA Filament Production

PLA and reprocessed PLA (PLA-RP) were extruded using a 3Devo Composer 350 extruder (3Devo, Utrecht, The Netherlands), starting from Ingeo™ Biopolymer 2003D PLA pellets (NatureWorks^®^, Minnetonka, MN, USA). The extrusion was carried out with a temperature profile of 185 °C, 190 °C, 190 °C and 185 °C (from die to hopper), and a screw speed of 7.0 rpm. After extrusion, the entire filament was processed into granulated material to obtain pellets using a SHR3D IT unit (3Devo, Utrecht, The Netherlands). A portion of this granulated material was set aside, then dried in a J.P. Selecta oven (Barcelona, Spain) at 40 °C for 48 h to avoid moisture content. The dried material was subsequently re-extruded under the same processing conditions, producing a second filament identified as PLA-RP, to simulate the valorization of PLA from industrial lines (defectives parts, edges, burrs and scraps produced during manufacturing).

#### 2.2.2. PLA Film Production

Films of neat PLA and PLA-RP were produced using a Mr. Hide Extracts WTRP-10T Rosin press (Tarragona, Spain). Previously spooled filaments were conditioned at 40 °C for at least two days prior to processing. Approximately 1.5 g of each shredded filament was placed between two metal plates, with non-stick sheets placed between the plates and the material. Various compression cycles were performed (aiming to eliminate trapped air bubbles [4]) at a set temperature of 200 °C, which corresponded to an actual temperature of approximately 160 °C due to heat losses between the plates. The process began with a 2 min preheating step, followed by pressing the material for 1 min at 3 MPa, then 2 min at 5 MPa. The pressure subsequently increased over 30 s to 30 MPa and was maintained for 5 min.

### 2.3. Characterization of the Materials

#### 2.3.1. Pellet Characterization

- Melt Flow Index

To assess the processability of the PLA and PLA-RP pellets, an MFI analysis was performed using a Metrotec MFI-100 analyzer (Techlab Systems, Lezo, Spain). The test was conducted at a fixed temperature of 190 °C with a 2.16 Kg load applied. Each sample underwent six iterations, with individual measurements lasting 15 s.

- Viscosity average molecular weight

The capillary viscosity of the virgin PLA and PLA-RP pellets, as well as that of the obtained films, was measured with a type 1C Ubbelohde viscometer at 25 °C using a water bath Selecta VB-1423 (J.P. Selecta, Abrera, Spain). Each sample was dissolved in CHCl_3_ and at least four concentrations were measured. The intrinsic viscosity [η] of each sample was determined and further used to estimate the average viscosity molecular weight using the Mark–Houwink relation (Equation (1)).

(1) $$\left[\eta \right]=K\times M_{v}^{a}$$

in which *K* and *a* for the PLA polymer are 1.53 × 10^−2^ and 0.759, respectively [8].

- Volatile, semi-volatile and non-volatile compounds

The study of non-intentionally added substances (NIASs) has become a key tool for evaluating potential contaminants that may arise during the reprocessing of materials.

An analytical approach was used to detect a broad spectrum of organic compounds in both virgin and reprocessed PLA pellets, including volatile (VOCs), semi-volatile (SVOCs) and non-volatile compounds (NVOCs). Headspace gas chromatography coupled with mass spectrometry (HS GC/MS), solvent extraction followed by GC/MS, and ultra-high-performance liquid chromatography combined with electrospray ionization and high-resolution mass spectrometry (UHPLC ESI HRMS) were employed for the analysis of VOCs, SVOCs and NVOCs, respectively. Toluene was used as an internal standard for VOC and SVOC quantification. For NVOCs, the extraction medium was analyzed after sample contact, with benzyl butyl phthalate and nimesulide serving as internal standards in positive and negative ionization mode, respectively. Compound identification in all cases was based on comparisons with mass spectral libraries.

- Specific Migration of Metals

For the reprocessed PLA pellet (PLA-RP), the presence of metals was also analyzed to ensure that no substances exceeded the specific migration limits (SMLs) established by Regulation (EU) No. 10/2011, assessing the potential release of elements related to the increased mechanical stress experienced during reprocessing. The evaluation was carried out using 3% acetic acid (simulant B) under immersion conditions at 60 °C for 10 days, with a surface-to-volume ratio of 6 dm^2^/kg. The elements analyzed included Ba, Co, Mn, Zn, Cu, Fe, Li, Al, Ni, Eu, Gd, La, Tb, As, Cd, Cr, Pb, Hg and Sb, following the UNE-EN 13130-1:2005 standard [18] and an internal procedure. The applied test conditions reflect standardized scenarios for long-term food contact at room temperature or below, including hot-fill processes or brief heating up to 100 °C.

#### 2.3.2. Film Characterization

- Viscosity average molecular weight

The capillary viscosity of the films obtained from virgin PLA and PLA-RP was determined using the same procedure as for the pellets for comparison.

- Attenuated total reflectance Fourier transform infrared (ATR-FTIR) spectroscopy

The identification of functional groups and potential molecular interactions present in the samples was performed using a Jasco FT/IR-4X spectrometer (Jasco, Easton, MD, USA) equipped with an ATR (attenuated total reflectance) accessory. Spectra were acquired using Spectra Manager 2005 software, with 64 scans recorded per sample over the 400–4000 cm^−1^ range, at room temperature, in transmission mode and with a resolution of 4 cm^−1^.

- Scanning electron microscopy

The microstructure of the films’ cross sections was observed by field emission scanning electron microscopy (FESEM) by means of a JEOL JSM-IT700HR microscope from Oxford Instruments (Tokyo, Japan). The film samples were previously frozen in liquid N_2_, cryo-fractured and sputtered with a thin layer of gold and palladium alloy in a Quorum Q150TS/E/ES unit (Quorum Technologies Ltd., East Sussex, UK) to achieve a conductive surface. Then, the film samples were observed with an accelerating voltage of 15 kV, and images were taken at 500× and 1000× magnifications.

- Thermogravimetric analysis

Dynamic thermogravimetric analysis (TGA) was performed in duplicate using the TGA analyzer SETARAM (Caluire, France). The onset degradation temperatures were determined from the TGA curve, while the maximum degradation temperatures were determined from the derivative thermogravimetry (DTG) curves, which represent the rate of mass loss as a function of temperature.

- Differential Scanning Calorimetry

Differential scanning calorimetry (DSC) analyses were conducted in a SETLINE DSC from SETARAM (Caluire, France). The DSC thermal cycles were carried out under a nitrogen atmosphere. The first heating DSC scan was conducted from 25 °C to 200 °C at a rate of 10 °C/min with the main objective of eliminating the thermal history. Then, the samples were cooled down to 25 °C at a rate of 10 °C/min. Finally, the second heating DSC scan was carried out from 25 °C to 250 °C at a rate of 10 °C/min. Measurements for each sample were performed in duplicate. The degree of crystallinity (χ_c_), obtained from the DSC thermograms is calculated through Equation (2).

(2) $$\chi_{c}=\frac{\Delta H_{m}-\Delta H_{cc}}{\Delta H_{m}^{0}}\cdot \frac{1}{W_{PLA}}100$$

where Δ*H*_m_ is the melting enthalpy, Δ*H*_cc_ is the cold crystallization enthalpy, ΔH_m_^0^ is the melting heat associated with pure crystalline PLA (93 J g^−1^) [1] and *W*_PLA_ is the weight fraction of the PLA in the blend formulation.

- Mechanical properties

Tensile testing measurements of PLA and PLA-RP films were conducted using a Shimadzu AGS-X-100N universal testing machine (Kyoto, Japan) equipped with a 100 N load cell and operated via TRAPEZIUM X software (1.5.4 version). The initial grip separation was 30 mm, the crosshead speed was set to 10 mm/min and test parameters were configured according to ISO 527-1:2019 for both films [19]. Dog-bone samples for tensile specimen 1BB according to ISO 527-2 [20] were prepared using a JBA electrohydraulic cutter (Instruments J. Bot SA, Cabrils, Spain). Young’s modulus (E), tensile strength (σ_r_) and elongation at break (ε_r_) were calculated from the resulting stress–strain curves, and average values were reported from seven films’ specimens.

- Static Water Contact Angle Measurements (WCA)

The surface wettability of the PLA and PLA-RP films was studied through static water contact angle (WCA) measurements using a standard goniometer (Ossila BV, Leiden, The Netherlands) equipped with a high-resolution camera and Ossila software 4.1.4 version) for WCA analysis. The tests were performed at ambient temperature by dispensing five ~10 µL drops of distilled water onto randomly selected regions of each film surface. Contact angles were recorded 30 s after droplet placement, and the mean WCA values were calculated for each formulation.

- Water Vapor Transmission Rate (WVTR)

The rate of water vapor transmission through the films was determined following the UNE 53097 standard [21], which specifies a gravimetric method for evaluating water vapor transfer in sheet materials using silica gel as a desiccant agent. Samples were placed in dedicated permeability cups, with an exposed surface area of 10 cm^2^ (A, cm^2^). The cups filled with 2 g of previously dried silica gel were stored in a desiccator maintained at 21.4 ± 0.5 °C with a relative humidity above 70%, established using a saturated potassium nitrate (KNO_3_) solution.

Each capsule was weighed every hour for the first 7 h and again at 24 h from the start of the test in order to plot the weight gain as a function of time (t, h). The analysis was performed in triplicate. The slope (n = g/h) of the linear portion of the resulting curve was used to calculate the WVTR according to Equation (1), expressed in g/day·cm^2^. The final value was then normalized to a film thickness of 100 µm using Equation (2).

(3) $$WVTR=\frac{24\cdot \;n}{A\cdot t}$$

(4) $$WVTR_{norm}=\frac{WVTR\cdot \;e}{0.1}$$

where *e* is the thickness of the film (mm).

- Oxygen Transmission Rate (OTR)

The oxygen permeability of each film was carried out at 23 °C using a Systech Illinois OxySense^®^ 8101e Oxygen Permeation Analyzer (Industrial Physics Product Integrity Ltd., Thame, UK). During testing, nitrogen was introduced into the lower half of the sample chamber, while pure oxygen (99.9% purity) was supplied to the upper half. The test was carried out on films with an exposure area of 50 mm^2^ in two cells until a steady-state oxygen transmission rate was achieved, with five values recorded for each cell. The output values were obtained using the Systech Illinois software v4.5.5, and the OTR.*e* values of duplicates were reported.

- Global Migration

Global migration testing was performed in accordance with the UNE-EN 1186 [22] regulation, which standardizes the evaluation of plastic materials intended for food contact. Film specimens (45 mm^2^) were immersed in 7.5 mL of 50% *v*/*v* ethanol solution, used as a fatty food simulant (food simulant D1) and maintained at 40 °C for 10 days in 20 mL glass vials. Prior to testing, the empty vials were dried at 105 °C to a constant weight and their masses were recorded. After the exposure period, the simulant was evaporated, and the vials containing the residues were dried again at 105 °C to a constant weight, after which their final mass was recorded to determine the global migration. All tests were conducted in triplicate to ensure data reproducibility and reliability.

- Disintegration Tests Under Composting Conditions

Disintegration tests under composting conditions were conducted according to UNE-EN-ISO 20200:2024 [23], which describes the laboratory-scale determination of the degree of disintegration of plastic materials under simulated composting conditions. The objective of this test was to evaluate the physical disintegration of PLA and PLA-RP over time, assessing whether they can naturally disintegrate and whether reprocessing affects the disintegratability rate of PLA, a key property in efforts to reduce plastic pollution.

To prepare the compost, a mixture was formulated with the following dry-weight composition: 18% sawdust, 13.5% rabbit feed, 4.5% commercial compost, 4.5% corn starch, 2.3% sugar, 0.5% urea and 1.8% oil. Finally, water was added to reach a total moisture content of around 50% (*w*/*w*).

Film samples measuring 15 × 15 mm^2^ were used. After recording their initial mass $\left(m_{i}\right)$ in a semi-micro AX125D analytical balance (OHAUS Europe GmbH, Nänikon, Switzerland), seven replicates of each film formulation were placed in mesh textile bags to facilitate handling, recovery and exposure to the compost medium [24]. The bags containing the samples were buried at a minimum depth of 6 cm in the compost inside perforated plastic containers acting as reactors. These composting reactors were placed in a ventilated oven at 58 °C for 25 days. Aerobic conditions were maintained by periodically mixing the composting material, and moisture was restored by adding water when necessary. The samples were retrieved at different times (days 1, 4, 7, 11, 14 and 21), and any adhering residues were removed from the films by washing with distilled water. The recovered films were further dried under vacuum for 40 °C during 24 h and re-weighed at each extraction time. The compost changes as well as the film samples were visually inspected for qualitative analysis and visual appearance was documented through photographs. The residual mass $\left(m_{r}\right)$ of the recovered films was quantitative determined, while the disintegration degree (%D) was calculated using Equation (3).

(5) $$\%D=\frac{m_{i}-m_{r}}{m_{i}}\times 100$$

#### 2.3.3. Statistical Analysis

A randomized experimental design was considered for the experiments. Data analysis was carried out using InfoStat 2018e version. This software was used to implement variance analysis. Fisher’s LSD method was applied to identify significant differences between samples. Differences were considered significant at *p* < 0.05.

## 3. Results and Discussion

### 3.1. PLA and PLA-RP Pellet Characterization

#### 3.1.1. Melt Flow Index

The MFI of the PLA and PLA-RP were determined to obtain insights into the degradation of PLA because of the thermal reprocessing procedure. Typical MFI values for PLA are between 6 and 11 g/10 min [6,25,26]. The MFI values of PLA and PLA-RP resulted in 6.55 g/10 min and 9.93 g/10 min, respectively. Similar values were previously observed by other authors. For instance, Silva et al. studied the reprocessing of PLA and observed an increase in the MFI from 6.0 g/10 min in neat PLA pellets to 9.2 g/10 min after one extrusion processing cycle [10]. On the other hand, Agüero et al. studied injection-moulded PLA parts subjected to up to nine reprocessing cycles and observed that the MFI increased from 6 g/10 min in PLA pellets to 10 g/10 min in the first reprocessing cycle, which was mainly maintained up to the third reprocessing cycle, concluding that the first processing cycle is not critical in PLA degradation [6].

#### 3.1.2. Viscosity Average Molecular Weight

The viscosity average molecular weights (*M_v_*) of virgin PLA and reprocessed PLA (PLA-RP) pellets were also determined to explore the degradation of the polymeric matrix due to the thermal reprocessing procedure and are reported on Table 1. The estimated *M_v_* of PLA showed a reduction of around 5.5% after the reprocessing thermal cycle. Similar findings were reported by Hidalgo-Carbajal et al., who studied the reprocessing of PLA recovered from 3D printing parts and observed that during the first three extrusion cycles, the reduction in the average viscosity molecular weight mass was only around 9% [5]. The decrease in the *M_v_* of reprocessed PLA (PLA-RP) is ascribed to chain scission induced by thermal degradation during melt-processing, primarily due to a hydrolysis process, which is intensified by the elevated temperatures used during the extrusion process [7,8,10]. The average viscosity molecular weight of the films was further reduced because of the compression moulding thermal process applied, but the reduction was around 6.4% in the neat PLA film and around 6.7% in the PLA-RP film, with respect to their starting pellets, indicating that each thermal process affects the molecular weight of PLA-based materials.

#### 3.1.3. Volatile, Semi-Volatile and Non-Volatile Compounds

In order to ensure the suitability of PLA for food contact applications, in compliance with Regulation (EU) No. 10/2011, it is essential to demonstrate that no hazardous substances will migrate into food [27]. However, during the recycling process, substances potentially harmful to human health may be generated due to repeated exposure to high temperatures.

According to their properties, substances can be classified in groups: volatile organic compounds, primarily responsible for off-odours in recycled plastics [28]; semi-volatile organic; organic non-volatile compounds; and inorganic substances. While many of these are intentionally added substances (IASs) to improve the properties of the polymer, the presence of NIASs in plastic materials remains a critical issue for food contact applications, as well as for ensuring that materials entering other plastic streams are not affected by cross-contamination. These substances may originate from consumer misuse or the presence of non-food-grade packaging in the same waste stream. Additionally, they can be derived from chemical components introduced throughout the recycling process itself, such as during washing or reprocessing, or from reactions between these components, leading to the formation of new substances [12,29,30].

Recent advancements in decontamination technologies, including super-cleaning and dissolution–precipitation processes, demonstrate potential for achieving the necessary purity standards. Notable industrial efforts, such as those by Styrenics Circular Solutions with Ineos Styrolution as a founding member [31] by means of the decontamination technologies it has developed like NGR [31], have made progress in enhancing PS recycling technologies. For polypropylene (PP), some of the most advanced public studies originate from the UK’s Waste and Resources Action Programme (WRAP) in its various phases [32,33]. Particular emphasis is placed on the Nextloopp project [34] led by Nextek Ltd. (London, UK), which has also contributed to previous initiatives and brings together companies across the plastic value chain to foster the development of decontamination technologies for food-grade postconsumer recycled PP. Meanwhile, for high-density polyethylene (HDPE), especially in the context of bottles, real-case studies involving postconsumer materials collected in collaboration with public and private entities have been reported [35,36]. However, regulatory approval remains an ongoing process, requiring further optimization and validation. For these reasons, it is essential to prevent the release of substances that could potentially compromise the recyclability and safety of other plastics intended for food contact. In the case of PLA, the migration of monomers and degradation products, such as oligomers, aldehydes, amines and other low-molecular-weight substances, which are more susceptible to migration, raises concerns about potential health risks.

**Figure 1:**
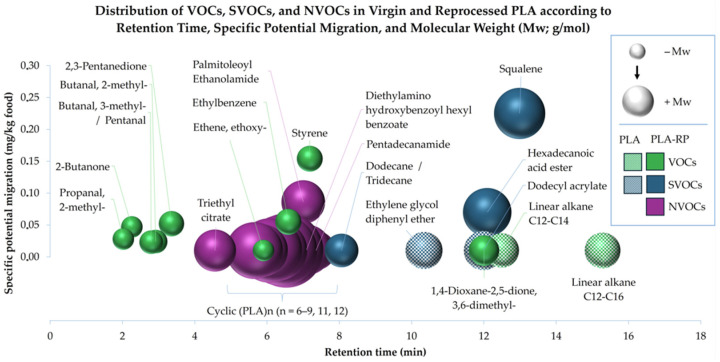
Distribution of VOCs, SVOCs and NVOCs in virgin and reprocessed PLA according to retention time (min), specific potential migration (mg/kg) and molecular weight (Mw; g/mol).

In the present study, a total of 28 substances were detected in reprocessed PLA, 26 of which were successfully identified based on spectral matching with reference libraries. Only two of these substances are currently listed in Regulation (EU) No. 10/2011. The potential specific migration was calculated using a surface-to-volume ratio of 6 dm^2^/kg [27]. A clear trend where non-volatile organic compounds (NVOCs), generally characterized by higher molecular weights, exhibited longer retention times and lower specific potential migration values can be observed in Figure 1. In contrast, most volatile organic compounds (VOCs), characterized by their lower molecular weights, eluted earlier. This highlights the difficulty decontamination technologies face in removing persistent substances like NVOCs due to their higher molecular weight and low diffusion rates [37,38] compared to volatiles, which are more readily removed, for example, through high-temperature vacuum degassing [39].

Notably, a distinct difference was observed between the virgin PLA and the reprocessed PLA (PLA-RP) samples. While most substances identified in PLA-RP eluted in the early part of the chromatographic window, the compounds detected in virgin PLA appeared predominantly at later retention times. This contrast may reflect subtle differences in the composition and structure of both materials. In PLA-RP, the predominance of earlier-eluting substances could be influenced by the presence of shorter PLA chains formed during thermal degradation, which may act as internal plasticizers, facilitating the release of embedded compounds [40]. Additionally, the formation of external contaminants during reprocessing may contribute to the presence of species that are typically more volatile and less strongly retained by the polymer matrix [41]. In contrast, the fewer substances detected in virgin PLA tended to elute later, which may be associated with stronger interactions with the polar PLA matrix or with their intrinsic lower volatility. Although the overall physicochemical properties of virgin and reprocessed PLA appeared similar, these results suggest that even minor structural or compositional changes can influence the retention and migration profile [42,43]. Importantly, all identified substances in both materials exhibited low specific migration values, supporting the limited release of compounds under the applied testing conditions.

Polarity also played a key role in the retention behaviour of the VOCs and SVOCs later in the chromatogram. Long-chain linear alkanes, such as hexadecanoic acid ester and squalene, are nonpolar compounds that, despite their classification, may have weaker interactions with the polar column, causing them to elute later. Moreover, their low polarity could also result in weaker interactions with the PLA matrix, favouring their release.

The studied PLA is composed of more than 98 wt% polylactide polymers (CAS 9051-89-2) according to the manufacturer [17], who also reports the inclusion of an external lubricant in the formulation [44]. These intentionally added substances (IASs) are relevant, as they are directly related to the formation of compounds detected as a result of the thermo-mechanical degradation of the materials.

Among all the compounds analyzed, *squalene* exhibited the highest specific potential migration, highlighting its elevated mobility despite its relatively high molecular weight. This behaviour is consistent with its highly nonpolar nature [45], which limits its interactions not only with the polar stationary phase used in the chromatographic separation but also with the PLA matrix itself, known to be polar due to its ester functional groups. As a result, squalene showed both delayed elution and high migration potential. This behaviour aligns with the wide use of squalane, a structurally related saturated analogue of squalene, as a reference compound for characterizing nonpolar stationary phases in gas chromatography, owing to its strong retention via dispersive interactions [46], highlighting how molecular structure and polarity in conjunction influence migration tendencies under the test conditions.

(a) Analysis of volatile organic compounds (VOCs)

Two *linear alkanes* (*C12–C16* and *C12–C14*) detected in the virgin PLA material, likely corresponding to linear oligomers, are commonly found in plant-derived food contact materials, i.e., agropolymers [47]. Aldehydes were the predominant functional group, including *2-methylpropanal* (CAS 78-84-2), *2-methylbutanal* (CAS 96-17-3) and *3-methylbutanal/pentanal* (CAS 590-86-3/110-62-3) (unresolved due to similar spectra and retention times). Acetaldehyde is the main thermal decomposition product of PLA, similar to polyethylene terephthalate (PET), and may participate in reactions leading to the formation of the detected substances [17]. These compounds are authorized as flavouring substances in food [48]; *pentanal* has been previously detected in corn starch trays and in PLA bio hot cup lids for serving beverages/soup [49], related to the biobased origin of PLA [50], including corn starch and sugarcane [51].

In PLA-RP, VOCs represented the majority of the detected substances and are particularly relevant as they are the main precursors of undesirable odours in recycled materials. *Styrene* (CAS 100-42-5) was the most abundant compound identified (PLA-RP = 0.154 mg/kg (154 ppb)), likely present due to cross-contamination from polystyrene residues in the extruder. Although Regulation (EU) No. 10/2011 does not establish a specific migration limit (SML) for styrene, EFSA stated in its 2025 re-assessment that a specific migration limit of 40 µg/kg food, proposed by the European Commission, “is not of safety concern” with respect to genotoxicity following oral exposure. EFSA also noted that for substances demonstrated to be non-genotoxic, an SML of up to 50 µg/kg food would not raise safety concerns [52]. However, higher limits were not within the scope of the evaluation. As styrene was not detected in the virgin PLA, its presence in PLA-RP underscores the importance of thorough cleaning and equipment segregation to prevent cross-contamination during PLA processing to ensure compliance with food contact regulations.

*Ethylbenzene* (CAS 100-41-4) may also originate from such cross-contamination, although its presence could equally be linked to its common use as a plasticizer in polyesters [53]. This explains its frequent detection in multiple recycled materials [42] and waste streams [54], being one of the reasons why it is often used as a standard surrogate in challenge tests to evaluate decontamination processes. Its detection in PLA-RP may also reflect a somewhat facilitated release due to reduced matrix interactions and structural changes associated with reprocessing.

Several compounds identified exclusively in the reprocessed PLA (PLA-RP) pellets, including *2,3-pentanedione*, CAS 600-14-6; *2-butanone*, CAS 78-93-3; and *1,4-dioxane-2,5-dione, 3,6-dimethyl-*, CAS 95-96-5 (a common form of lactide), have also been previously detected as volatile emissions during 3D printing using PLA filaments [51,55]. Their presence in PLA-RP is consistent with the secondary thermal degradation pathways of PLA, particularly involving lactide and short-chain oligomers, which could account for the formation of such low-molecular-weight compounds.

While these compounds were absent or below detection limits in the virgin PLA, their formation in PLA-RP reflects structural changes induced by reprocessing. Notably, ageing has also been shown to reduce VOC content over time [47] which may influence their detection depending on storage history.

Some VOCs, including ethyl vinyl ether (*ethene*, *ethoxy-*, CAS 109-92-2), were detected at levels below 0.1 mg/kg. This substance is commonly used in polymer production and copolymerization processes [45]. Its presence as an NIAS in PLA-RP may result from thermal degradation of ether-containing additives or cross-contamination during previous processing. Due to its high volatility and reactivity, even trace residues may persist and be released upon re-extrusion. Overall, only two of the volatile compounds detected could not be identified.

(b) Analysis of semi-volatile organic compounds (SVOCs)

Two substances were detected exclusively in the virgin PLA. *Dodecyl acrylate* or *Lauryl acrylate* (CAS 2156-97-0), found at levels below <0.01 mg/kg food, was the only compound identified in this study that is listed in Regulation (EU) No 10/2011, with a specific migration limit of 0.05 mg/kg food. The second compound, *Ethylene glycol diphenyl ether* (CAS 104-66-5), is an aromatic–aliphatic ether previously reported in polyester-based materials such as PET [53]. It is known to be used as an intermediate in the synthesis of polyesters [56], and Eckardt et al. used it as reference substance in the development of a quantification method for determination of oligomers from polycondensate plastics like PET and polyamides (PAs) [57]. As a phenol derivative, its presence may be attributed to processing aids used during PLA production or impurities by cross-contamination since it is also used in inks [58]. Therefore, it can be considered an NIAS as it is not included in the positive list of additives authorized for food contact materials. Its specific migration potential, being in PLA-RP in quantities of <0.01 mg/kg food, corresponding to the method’s limit of quantification, is well below the threshold of toxicological concern (TTC) according to the Cramer classification and, consequently, is not associated with a genotoxicity concern. The fact that it was detected in virgin PLA suggests it may originate from residual processing aids or cross-contamination, in line with the definition of NIASs, which also encompasses non-intentionally added substances present in virgin polymers.

Meanwhile, in PLA-RP, *squalene* (CAS 111-02-4) may be associated with contamination occurring during handling or recycling processes involving food contact materials from agropolymers. Although not intentionally added to PLA, it is a natural triterpene commonly found in vegetable oils such as amaranth and olive oil [59] and was previously studied for its toughening effect in PLA, despite its low solubility in the polymer matrix [60]. It has also been reported to migrate from wood-based and plant residue packaging [59], indicating a potential origin from biomass-derived components. Furthermore, it has been identified in various packaging materials, including PE/PET, PP/PP and PE/EVA films [61].

*Hexadecanoic acid ester*, also known as 2-ethylhexyl palmitate (CAS 29806-73-3), was also detected in PLA-RP. This compound has multiple industrial applications, including as a processing aid in lubricants and greases and as an additive in polymer formulations [62]. While it can be intentionally added in other polymer systems, in this case, its presence is more likely due to contamination or transfer from processing aids. As it is not included in the positive list of additives authorized for food contact materials under Regulation (EU) No 10/2011, it is considered an NIAS. Its potential specific migration was estimated at 0.070 mg/kg food, which is below the threshold of toxicological concern (TTC) according to the Cramer classification and, therefore, is not associated with a genotoxicity concern.

Also found exclusively in PLA-RP was the aliphatic hydrocarbon *dodecane* (CAS 112-40-3), which, due to its structural similarity and overlapping retention times, may alternatively correspond to *tridecane* (CAS 629-50-5) [28]. Dodecane is also classified by EFSA as a flavouring substance and occurs naturally in butter, tea and meats. It is capable of forming innocuous metabolites and belongs to structural class I, with a threshold of toxicological concern of 1800 µg/person/day [63,64]. In plastic packaging, dodecane and related hydrocarbons have been reported as NIASs in recycled polyolefins and are typically associated with the polymer backbone rather than with specific additives or contaminants [42,65]. Its exclusive detection in PLA-RP may be attributed to thermal degradation, environmental exposure or prior contamination from mixing or contact with polyolefin materials during recycling.

(c) Analysis of non-volatile organic compounds (NVOCs)

It must be highlighted that the virgin PLA did not reveal the presence of non-volatile compounds. In the case of the reprocessed PLA (PLA-RP), six different non-volatile oligomers were detected (*Cyclic (PLA) 6,7,8,9,11,12*), along with four identified substances.

*Palmitoleoyl Ethanolamide* (CAS 94421-67-7) was detected with a potential specific migration of 0.086 mg/kg of food. This ethanolamide, derived from palmitoleic acid and classified as a *N*-acylethanolamine (NAE) [45], has not been commonly reported as a contaminant in PLA. However, its presence in reprocessed PLA suggests it may originate from chemical reactions occurring during thermal processing. Importantly, palmitoleic acid itself is listed as an authorized substance (CAS 373-49-9) under Regulation (EU) No 10/2011 for use in plastic materials intended to come into contact with food [27], where it may serve as an additive, surfactant or adhesive [66]. This further supports the plausibility of its ethanolamide derivative appearing as a minor byproduct in recycled PLA formulations.

*Diethylamino hydroxybenzoyl hexyl benzoate*, or DHHB, (CAS 302776-68-7) in PLA likely originates from upstream sources, such as shared recycling equipment or intentional UV stabilization in the original polymer formulation, due to its function as an organic UVA filter [67].

*Pentadecanamide* (CAS 3843-51-4) was detected in the reprocessed PLA sample. While PLA itself does not contain nitrogen in its polymer backbone, the presence of this amide suggests the involvement of external nitrogen sources, possibly introduced through additives or via contamination with nitrogen-containing polymers, such as polyamides [68]. These components may contribute to the formation of minor byproducts like pentadecanamide during high-temperature processing.

Finally, the branched aliphatic ester *triethyl citrate* (CAS 77-93-0) was detected at levels below 0.01 mg/kg of food. This compound is commonly used as a plasticizer in food contact materials, suggesting that its presence may originate from such intentional use [69,70].

Although both virgin and reprocessed PLA may contain comparable residual moisture (i.e., absorbed during handling), in virgin PLA, the water is not exposed to high melt-processing temperatures capable of triggering ester bond cleavage [71]. As a result, no hydrolytic degradation products were detected in virgin PLA under the applied analytical conditions. In contrast, during melt reprocessing in PLA-RP, residual moisture can promote hydrolysis, producing shorter chains with hydroxyl and carboxyl end groups [71]. These low-molecular-weight species may subsequently undergo intramolecular transesterification reactions, also known as backbiting, leading to the formation of cyclic oligomers such as lactide and higher cyclic (PLA)_n_ species [71], detected exclusively in PLA-RP. The observed 5.5% decrease in viscosity average molecular weight further supports this mechanism, which also explains the occurrence of low-molecular-weight aldehydes and ketones (e.g., 2-butanone, 2-methylpropanal, 2,3-pentanedione, methylbutanals) through secondary thermo-oxidative degradation of hydrolytically shortened chains [47].

Despite the overall low migration values observed, certain substances identified in the reprocessed PLA exceeded the threshold of toxicological concern (TTC) for genotoxicity (0.00015 mg/kg food), notably including *2-methylpropanal* (0.028 mg/kg food), *3-methylbutanal/pentanal* (0.023 mg/kg food) and *diethylamino hydroxybenzoyl hexyl benzoate* (0.011 mg/kg food). The TTC approach is used when the structure of a substance is known but there is little specific toxicity data, allowing its safety to be estimated based on that structure [72,73]. According to EFSA, no data or genotoxicity concerns have been reported for these substances in the Chemical Hazards Database (OpenFoodTox) as of 2021, with the information reviewed in 2023 [64]. Regarding their safety and applications, only limited studies have been found. For instance, Chen et al. reported an average concentration of 0.03215 mg/kg of *2-methylpropanal* in 28 flavoured milk samples [74], while EFSA identified 0.9 mg/kg of *3-methylbutanal* in a smoke-flavoured primary product SF-005 [75]. However, no toxicological concern was raised for this compound, and the overall safety concern was attributed to other substances in the mixture.

A lack of data has been found with regard to the exposure and toxicity of *diethylamino hydroxybenzoyl hexyl benzoate* (DHHB) in the context of oral exposure and human consumption. The only available evaluation applies to cosmetic products, where the Scientific Committee on Consumer Products (SCCP) concluded that its use at concentrations up to 10% *w*/*w*, including in sunscreens, does not pose a risk to consumer health. However, due to limited data on exposure via other routes, the safety of such applications could not be fully assessed [76]. These findings underscore the importance of monitoring NIASs in reprocessed bioplastics, even those with industrial origins and controlled feedstock. While most substances likely derive from thermal degradation or processing additives, the appearance of new low-molecular-weight compounds or cross-contaminants during reprocessing may impact safety [30]. Thus, the implementation of effective decontamination steps, robust analytical screening and alignment with regulatory thresholds are critical for ensuring that reprocessed PLA remains a viable option for food contact applications.

#### 3.1.4. Specific Migration of Metals

The elemental analysis of the reprocessed PLA sample (PLA-RP) revealed that none of the tested metals exceeded their respective specific migration limits (SMLs) established in Regulation (EU) No. 10/2011 [27]. The presence of metals in recycled polymers has been previously attributed to contamination from additives, pigments, stabilizers, catalysts, coatings or external sources during waste collection and reprocessing, as supported by recent studies on recycled polyolefins and mixed plastic feedstocks [77,78]. Following the UNE-EN 13130-1:2005 standard under worst-case exposure conditions, all measured values were below the corresponding limits of detection, confirming the absence of detectable metal migration under the applied testing scenario [77]. The analysis was conducted exclusively on the reprocessed material, as the virgin PLA was not expected to contain trace metals based on the specifications provided by the supplier. This outcome suggests that the reprocessing conditions applied to PLA did not induce a significant release or formation of metal residues, supporting the chemical stability of the reprocessed PLA and indicating that the use of reprocessed PLA feedstock under the studied conditions would not compromise its compliance with EU food contact legislation in this aspect.

This outcome suggests that the reprocessing conditions applied to PLA did not induce the significant release or formation of metal residues.

### 3.2. Neat PLA and PLA-RP Film Characterization

#### 3.2.1. Attenuated Total Reflectance Fourier Transform Infrared Spectroscopy

For the spectra, the characteristic absorption bands of PLA were observed. The peaks at 754 cm^−1^ and 867 cm^−1^ are linked to the crystalline and amorphous phases of PLA, respectively, and correspond to C–C stretching vibrations [79]. Bands at 1043 cm^−1^, 1080^−1^ and 1180 cm^−1^ are assigned to C–O single bond stretching. The three bands are associated with CH_3_ bending and the symmetric and asymmetric C–H vibrations of the –CH_3_ groups in the PLA structure are identified at 1360 cm^−1^, 1380 cm^−1^ and 1452 cm^−1^ [80]. The strong band at 1747 cm^−1^ is attributed to the amorphous ester carbonyl (C=O) stretching vibrations [81,82]. The double peak at 2995 cm^−1^ and 2946 cm^−1^ corresponds to the asymmetric and symmetric stretching vibrations of the axial –CH groups in saturated hydrocarbons (–CH_3_) [79].

**Figure 2:**
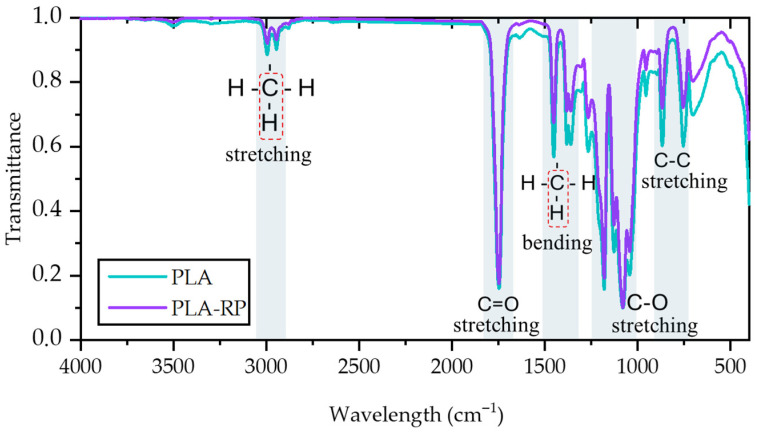
Spectra of neat and reprocessed PLA samples. The shaded blue areas highlight the main characteristic bands of PLA and PLA-RP, while the red dotted boxes indicate C–H vibrations of –CH_3_ groups.

In Figure 2, a small difference in the intensity of the spectral bands between approximately 700 and 2000 cm^−1^ can be observed when comparing neat and reprocessed PLA. For instance, there was a band centred at 956 cm^−1^, assigned to PLA’s amorphous phase [10], that shows a slightly higher intensity in PLA than in PLA-RP, while the band centred at 920 cm^−1^, corresponding to the 10_3_ helix chain conformation and characteristic of crystalline PLA [83], practically did not appeared in both materials. These small variations have been associated with structural changes resulting from reprocessing, such as chain scission or a reduction in molecular weight, which can alter the IR absorption behaviour of the material [84]. Silva et al. studied the reprocessing of PLA after a simulated postconsumer process (immersion of PLA in food simulants before reprocessing) and observed that there were no significant changes in the FTIR spectrum at the end of the reprocessing. They ascribed this behaviour to the fact that the reprocessing did not significantly affect the PLA’s polymeric structure [10].

#### 3.2.2. Scanning Electron Microscopy

**Figure 3:**
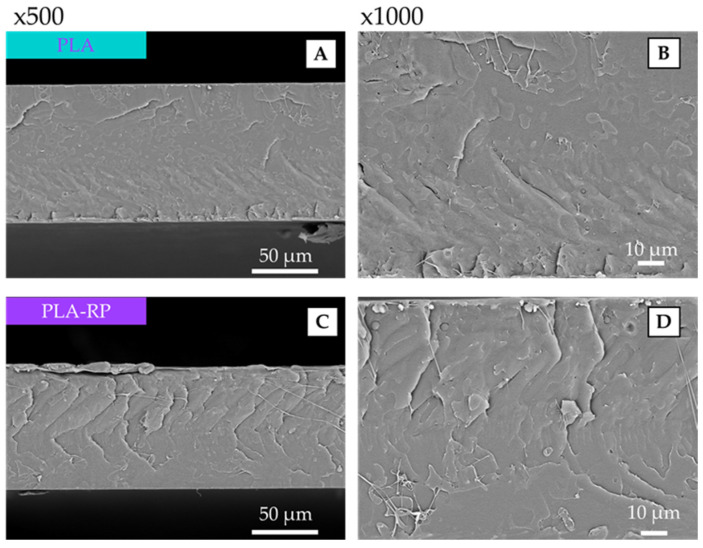
SEM images of neat PLA (**A**,**B**) and PLA-RP (**C**,**D**).

The morphological characterization of the cross-cryo-fractured sections of PLA and PLA-RP films was performed by SEM analysis, and the obtained micrographs are shown in Figure 3. The virgin PLA film (Figure 3A,B) shows a typical uniform and smooth surface of an amorphous polymeric matrix already observed in neat PLA films [4,85,86]. A very similar pattern was observed on the microstructure of PLA-RP (Figure 3C,D), showing a somewhat tougher cryo-fractured surface, probably due to the rather increased crystallinity in reprocessed PLA. Badia et al. studied the reprocessing of PLA and observed microstructural changes by SEM after five reprocessing cycles [7]. Similarly, Nomadolo et al. reported comparable findings in the microstructure of reprocessed PLA, even after the sixth cycle [87].

#### 3.2.3. Thermogravimetric Analysis

**Figure 4:**
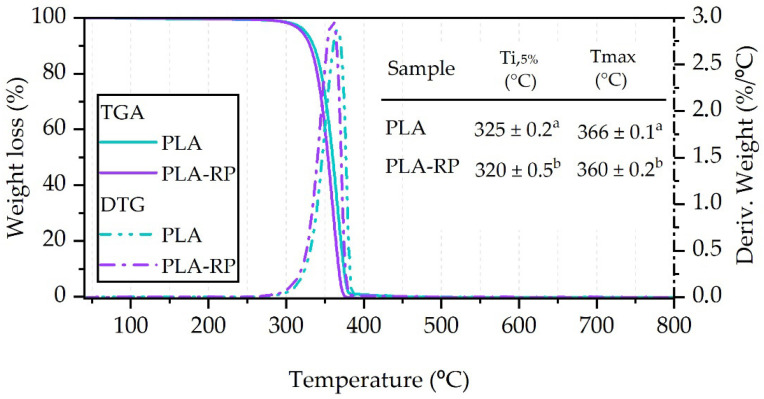
Dynamic TGA and their derivative (DTG) curves, TGA thermal parameters onset degradation temperature (T_i,5%_) and maximum degradation temperature (T_max_) of neat PLA and PLA-RP. Identical lowercase letters above the bars indicate no significant difference between samples (*p* > 0.05).

Figure 4 shows the weight loss (TGA) and derivative (DTG) thermograms of the PLA films obtained from the neat PLA and the PLA-RP. As expected, after the thermo-mechanical reprocessing cycle, the PLA-RP film showed a decrease in the thermal stability of PLA. The onset degradation temperature (T_i,5%_) decreased by 5 °C from 325 °C in the neat PLA film to 320 °C in the PLA-RP film, while the maximum degradation temperature decreased by 6 °C from 366 °C in the neat PLA film to 360 °C in the PLA-RP film, which is associated with PLA thermo-mechanical degradation [10]. These results are in good agreement with the already commented reduction in the molecular weight when discussing the intrinsic viscosity measurements, as the shorter polymeric chains degrade at lower temperatures. Similarly, the maximum degradation temperature (T_max_) value decreased by 6 °C. Similar reductions (around 5 °C) were observed in both the T_i,5%_ and T_max_ temperatures by Agüero et al. after one reprocessing cycle [6], and they further studied successive reprocessing cycles and observed that T_5%_ and T_max_ remained nearly constant, concluding that the reprocessing cycles do not have a major effect on thermal degradation temperatures of PLA.

#### 3.2.4. Differential Scanning Calorimetry

**Figure 5:**
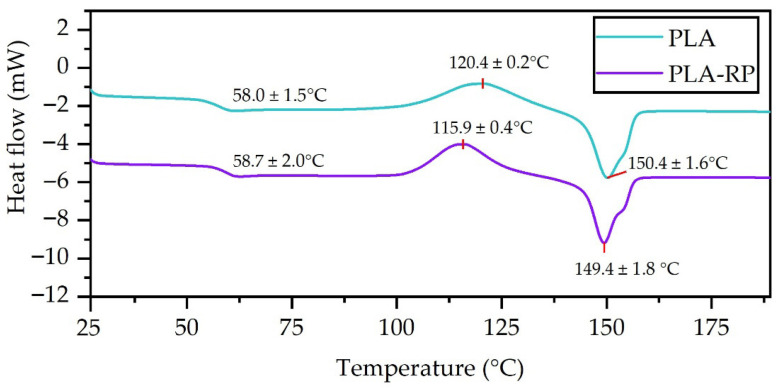
DSC thermograms from the first DSC scan of virgin and reprocessed PLA (10 °C/min).

DSC thermograms of the PLA- and PLA-RP-based films obtained from the first heating scan are reported in Figure 5, while the thermal properties, glass transition (*T*_g_), cold crystallization exothermic peak (T_cc_) and melting endotherm (T_m_), are summarized in Table 2. The T_g_ was practically the same in PLA and PLA-RP. Similarly, Carrasco et al. studied PLA and reprocessed PLA by injection moulding and observed that the T_g_ was approximately the same for processed and reprocessed materials; they concluded that crystal thermodynamic stability (related to crystal thickness) was not affected by the reprocessing step [88]. Silva et al. studied the reprocessing of simulated postconsumer PLA and observed a reduction in the T_g_, but only after five reprocessing cycles, and ascribed that behaviour to the presence of shorter PLA chains that act in plasticizing the system [10]. The lack of a reduction in the T_g_ in PLA-RP suggests that a non-high amount of shorter polymer chains is present due to the low degradation of PLA, as was observed by the small reduction in the M_v_ and slight increase in the MFI of PLA. On the other hand, the cold crystallization enthalpy (ΔH_cc_) resulted in higher values in PLA-RP than in the neat PLA film, suggesting that reprocessing modifies the crystallization kinetics of PLA [7]. Similarly, PLA-RP shows a cold crystallization temperature about 5 °C lower than the neat PLA. This small reduction in T_cc_ could be attributed to some thermo-mechanical degradation of PLA during reprocessing because shorter polymer chains increased their mobility and were more ready to pack in an ordered structure at lower temperatures and thus act as nucleation centres, which led to increased crystallinity [6,7]. Regarding the T_m_, a melting enthalpy (ΔH_m_) was observed, ascribed to the melting of less-ordered crystalline domains at lower temperatures [83]. Nevertheless, it should be highlighted that these are minor changes. Finally, PLA-based films showed a very low crystallinity degree, evidencing that both PLA and PLA-RP are essentially amorphous, in good agreement with the visual aspect of the highly transparent obtained films. The low crystallinity obtained can be attributed to the residual crystallinity of the PLA used in this work (4.5% content of D-isomer) as it is known that PLA containing more than 93% L-lactic acid units is usually amorphous, therefore confirming its poor ability to crystallize [3,89]. The similar enthalpy values for the cold crystallization and subsequent melting observed in the neat PLA film indicate that its crystallization exotherm and melting endotherm had similar heat contents; therefore, the quenching applied after the compression moulding process avoided the crystallization of PLA, allowing the production of amorphous films. Although low, the PLA-RP film showed somewhat higher crystallinity. Therefore, it can be concluded that the reprocessing did not substantially induce crystallization of PLA, remaining mostly amorphous after reprocessing. It should be highlighted that although PLA containing more than 93% L-lactic acid units can crystallize, it is usually amorphous, as its high molecular weight reduces the crystallization rate and, thus, the crystallinity degree [89], confirming that the reduction in the viscosity average molecular weight observed in this work due to the reprocessing did not substantially affect the crystallization of PLA during film processing.

#### 3.2.5. Mechanical Properties

**Figure 6:**
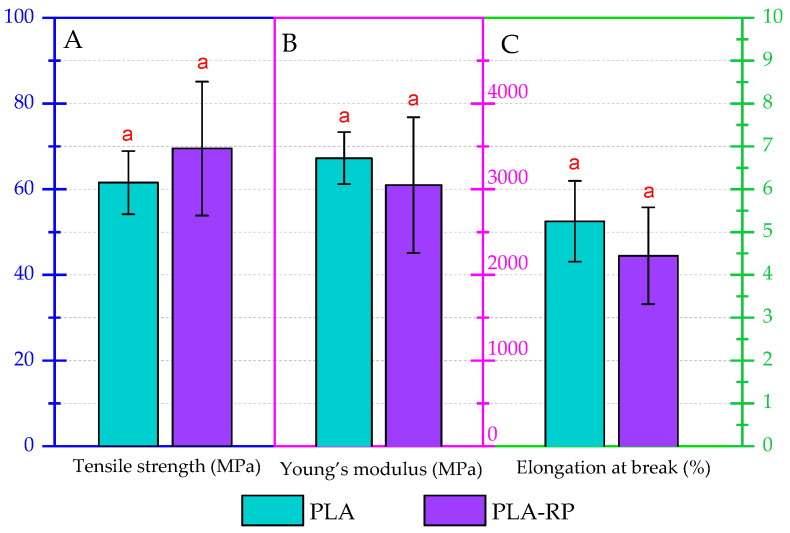
Tests on tensile properties of PLA and PLA-RP films: (**A**) tensile strength, (**B**) Young’s modulus and (**C**) elongation at break for virgin and reprocessed PLA. Identical lowercase letters above the bars indicate no significant difference between samples (*p* > 0.05).

The mechanical properties of the neat PLA and PLA-RPG films were evaluated by means of tensile test measurements. Although the overall mechanical properties showed scattered values with somewhat of a reduction in the tensile strength, Young’s modulus and elongation at break, these changes were not significantly different (Figure 6). It should be highlighted that the reprocessing of PLA has been widely studied, and it was observed that the tensile test properties remained almost unaffected during the first two reprocessing cycles [7,88]. The results suggest that although a small reduction in the average viscosity molecular weight was observed, it did not significantly affect the mechanical performance of PLA-RP, as its mechanical properties are mainly maintained.

#### 3.2.6. Static Water Contact Angle Measurements (WCA)

As PLA is very sensitive to water, the surface wettability of virgin and reprocessed PLA (PLA-RP) films was evaluated through static water contact angle (WCA) measurements. As shown in Table 3, the neat PLA film exhibited a contact angle of 74.6 ± 1.4°, while the PLA-RP sample showed a nearly identical value of 74.7 ± 1.3° (without significant differences), indicating that the reprocessing did not significantly affect the surface hydrophobicity of the PLA-based material. These values are slightly higher than those commonly reported for neat PLA films in the literature. For instance, Galvez et al. (2020) reported a similar contact angle of 70.1° for PLA films processed by melt extrusion and compression moulding [90]. A comparably higher angle of 73.0° was found by Agüero et al. (2023) for neat PLA films obtained by melt extrusion followed by a film forming process [85]. The small differences in WCA values obtained here with respect to those obtained in the literature are attributed to differences in film preparation techniques, which may change the surface porosity and regularity, as the wetting properties are affected by the topography of the material. The absence of a statistically significant difference between PLA and PLA-RP indicates that the reprocessing process does not significantly alter the surface-wetting behaviour of both studied PLA-based films (neat PLA and PLA-RP). It should be noticed that both formulations showed a hydrophobic surface as they showed values higher than 65° [91], being materials acceptable for the intended end-use applications in the food packaging field. This finding is particularly relevant for sustainable packaging applications, as it suggests that surface-dependent interactions such as wetting, coating adhesion and water repellence can be preserved in reprocessed PLA materials without the need for other additives or further surface modifications.

#### 3.2.7. Water Vapor Transmission Rate (WVTR)

The water vapor transmission rate (WVTR) test for neat PLA and PLA-RP films was carried out at 21.4 ± 0.5 °C and 70% RH via a gravimetric method based on the UNE 53097 standard [21], and the results are shown in Table 3. The neat PLA film exhibited a WVTR of 31.2 ± 1.1 g/m^2^·day, whereas the PLA-RP film showed a somewhat but non-significant increased value (increased of 2.7 ± 1.9 g/m^2^·day). Similar WVTR values have been previously reported in the literature. For instance, Sahoo et al. reported a WVTR of 172 g/m^2^·day for neat PLA films under lower humidity (50% RH, 23 °C) [92]. Meanwhile, Tee et al. obtained WVTR values of 23.1 ± 0.5 g/m^2^·day for neat PLA manufactured by melt hot pressing and measured in similar conditions with somewhat higher humidity (37.8 °C and 90 ± 2% RH [93], according to ASTM F1249-06 [94]). The WVTR values measured in this study are in the intermediate range of those previously reported for PLA. In particular, the minimal increase in WVTR observed in PLA-RP suggests that the reprocessing cycle does not produce a high amount of shorter polymer chains able to increase the polymer chain mobility and facilitate the diffusion of moisture throughout the film, thus preserving the water barrier’s functional integrity, which is very important for packaging applications.

#### 3.2.8. Oxygen Transmission Rate (OTR)

Films intended for food packaging must act as effective barriers against oxygen since oxygen exposure can accelerate spoilage, oxidation of fats and loss of the nutritional and sensory quality of packed food. Measuring the oxygen transmission rate (OTR.*e*) is therefore essential to evaluate the protective performance of PLA-RP films for food packaging applications. General OTR.*e* values for neat PLA are between 20 and 30 cm^3^·mm/m^2^·day [3,95,96]. As observed in Table 3, the reprocessed film’s value, with 21.6 cm^3^·mm/m^2^·day, is about two units higher than that for neat PLA (19.6 ± 1.3 cm^3^·mm/m^2^·day), indicating a marginal decrease in the barrier properties of the material due to the reprocessing step. Similarly, Auras et al., who studied the oxygen barrier properties of oriented PLA with 40% recycled content from the industrial trimming process, observed an increase in the oxygen permeability coefficient (89% increase) and concluded that the incorporation of recycled contents of PLA to neat PLA provides an opportunity for full material utilization at lower costs despite a reduced oxygen barrier [1]. Burgos et al. studied PLA plasticized with 15, 20 and 25 wt.% of oligomeric lactic acid (OLA) and observed an increase in oxygen transmission due to the plasticization effect of OLA (increment between 29% and 93% with increasing plasticizer content) accompanied by a reduction in the T_g_, and ascribed that reduction in oxygen barrier performance to the increased polymer chains’ mobility [3]. They further studied the ageing of PLA and PLA-OLA films for 90 days, and no significant differences in OTR.*e* values were detected in samples with the lower amount of OLA of 15 wt.% [3]. In the present work, the small reduction in the OTR.*e* value could be ascribed to somewhat of an increase in the polymer chain mobility produced by shorter polymer chains present in the reprocessed PLA film (PLA-RP) that provoked an increase in the free volume of the PLA matrix and, thus, allowed higher mobility of the oxygen molecules through the polymeric matrix. The smaller reduction observed here could be directly related with the non-significant reduction in the T_g_ value, which demonstrates the low amount of shorter polymeric chains present in the PLA-RP film sample. Moreover, it should be highlighted that although the OTR.*e* value of PLA increased with the reprocessing step, the OTR.*e* values of both the neat PLA film and reprocessed PLA counterpart (PLA-RP) were still better than the levels obtained for typical polymers used for food packaging, such as LDPE films (OTR.*e* around 160 cm^3^·mm/m^2^·day) [3,4].

#### 3.2.9. Global Migration Test

Although the analysis of volatile, semi-volatile and non-volatile compounds, as well as the specific migration of metals, showed the potential of reprocessed PLA for food contact applications, overall migration tests in fatty food simulants were carried out to determine the total amount of non-volatile substances that might migrate into foodstuff from PLA and PLA-RP films.

**Figure 7:**
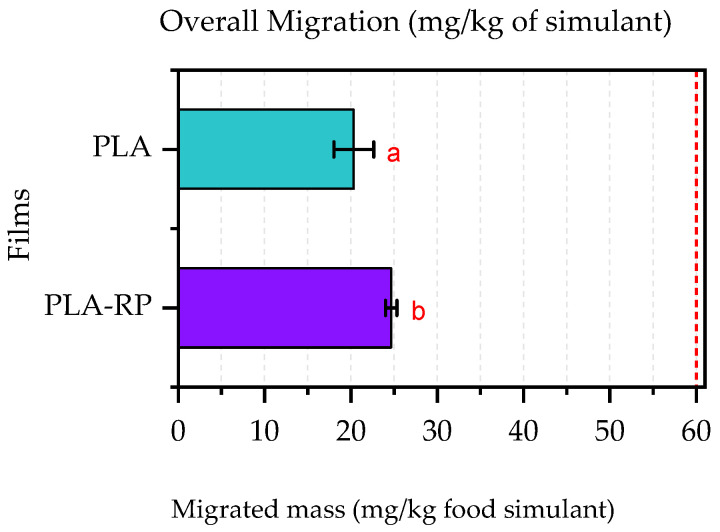
Overall migration of PLA and PLA-RP films in food simulant D1 (ethanol 50% *v*/*v*). The red line indicates the overall migration limit (60 mg/kg of food simulant) according to the directive UNE-EN 1186. Lowercase letters a–b indicate significant differences among the values between different materials (*p* < 0.05).

The overall migration results of PLA and PLA-RP into the food simulant D1 after 10 contact days are shown in Figure 7. The results show that PLA-RP presents some higher migration levels (around 4 mg/kg more of food simulant). It is known that the polarity and solubility properties of polymers play an important role on migration from packaging to foodstuff due to interactions between the food simulant, the polymeric film and the migrants [97]. In the present work, somewhat increased migration was observed in the PLA-RP film compared to the neat PLA film since the food simulant might be absorbed in the PLA-RP polymeric matrix, leading to somewhat of a release of the PLA degradation products formed during reprocessing. Nevertheless, it should be highlighted that both materials resulted in migration levels well below the legal overall migration limit of 60 mg/kg of food simulant, thus demonstrating a non-migrating behaviour for PLA-RP, and thus, its possible practical application in the food packaging sector [27].

#### 3.2.10. Disintegration Under Composting Conditions

**Figure 8:**
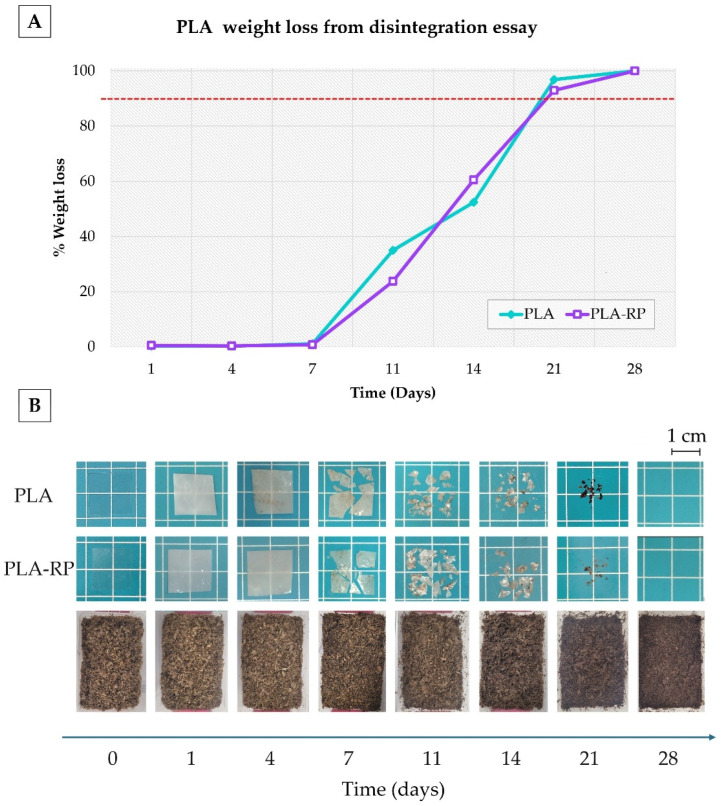
(**A**) Disintegration degree of PLA and PLA-RP films under composting conditions as a function of time. The red dashed line represents the 90% disintegration threshold. (**B**) Visual appearance of PLA and PLA-RP films before and after different incubation days under composting conditions.

As each thermo-mechanical reprocessing cycle negatively affects the thermo-mechanical performance of the PLA matrix, PLA products could not be infinitely recycled and composting them is the final end-life option [9,83]. Therefore, the films’ disintegratability was evaluated, according to Equation (5), in terms of mass loss as a function of composting time (Figure 8A), while the visual appearance of recovered PLA and PLA-RP films at different times of composting disintegration was qualitatively evaluated by taking photographs (Figure 8B). The films showed practically the same disintegration rate (Figure 8A), confirming that the material, structurally, did not significantly change.

From the visual aspect of the films (Figure 8B), it can be seen that in only one day under composting conditions, the films lost their transparency and became white, in good accordance with previous works [24]. The PLA-based films’ colour changed during the first stages of the laboratory-scale composting disintegration test (between 1 and 4 days), and they were ascribed to the beginning of the hydrolytic degradation process, making some changes in the films’ refraction index due to water absorption and/or the presence of hydrolysis products [86]. Moreover, along with the compost assay, both films increased their opacity. The apparent loss of transparency in the PLA and PLA-RP films during their exposure to a composting medium was due to enzymatic actions of microorganisms that can attack the shorter polymer chains formed at the initial stage of this disintegration process in the amorphous phase of the polymer [24]. After 7 days under the composting medium (Figure 8A), both the PLA and PLA-RP films started to become breakable, suggesting that the hydrolysis process was started.

It is worth noting that both PLA-based films were totally disintegrated under composting conditions in less than three weeks as they reached 90% mass loss, the goal of disintegratability tests frequently used in the literature [24,98,99]. In Figure 8B, it can also be seen the evolution of the compost soil during the PLA and PLA-RP disintegration, which finally results in humus-rich soil due to the aerobic fermentation of thermophilic bacteria.

## 4. Conclusions

Neat PLA pellets were subjected to a reprocessing extrusion cycle (PLA-RP), aiming to simulate the revalorization of PLA waste from industrial PLA products rejected during the production line (discarded defective PLA parts, plastics edges, burrs, scraps, etc.), and were evaluated as food contact material. The average viscosity molecular weight decreased around 5.5% due to reprocessing, while the MFI increased by 52%, indicating that reprocessing produced some chain scission, which led to shorter polymer chains with increased mobility. The presence of non-intentionally added substances (NIASs) was also assessed, and in both the neat PLA and reprocessed PLA (PLA-RP), all substances with specific migration limits remained below applicable thresholds, including trace metals. In neat PLA, fewer compounds were detected, likely related to residual additives. A higher variety and concentration of volatile and non-listed compounds was observed in reprocessed PLA (PLA-RP). Most identified substances are typically linked to thermal degradation, additives and special contaminants introduced during reprocessing, with three exceeding their assigned Cramer class thresholds, requiring further toxicological assessment. This reinforces the importance of combining migration testing with structure-based toxicological screening when evaluating reprocessed PLA for food contact applications.

As reprocessed PLA shows great potential as a non-migrating material, films were prepared from PLA-RP pellets by compression moulding and fully characterized. Films from neat PLA pellets were also prepared for comparison.

The obtained films showed that the structural and mechanical properties were mainly maintained in reprocessed PLA films (PLA-RP), and the changes in their thermal parameters were not remarkable. While the reprocessing of PLA did not provoke major changes in OTR.*e* values but showed somewhat of a decrease, both the surface wettability and water vapor barrier properties of reprocessed PLA (PLA-RP) remained comparable to those of neat PLA films, confirming that the reprocessing of PLA does not significantly affect its wetting and water diffusion properties. Finally, the disintegration test under composting conditions confirms that PLA mainly maintains its disintegrable character after the reprocessing.

The results obtained in this study show that reprocessed PLA holds great potential as a low-migration material of interest for the development of sustainable food packaging. Nonetheless, further validation through toxicological assessment remains essential to confirm its suitability for specific food contact uses, as safety may vary depending on the nature of the food, contact conditions and the potential exposure to individual substances. Controlling cross-contamination throughout the process, i.e., using an extruder only for PLA and its reprocessing and ensuring proper decontamination, are also key to mitigating the presence of non-intentionally added substances and ensuring consumer safety.

## Figures and Tables

**Table 1:** Viscosity average molecular weight of PLA and PLA-RP as well as that of their films.

Sample	*M_v_* (g/mol)	MFI (g/10 min)
PLA pellet	113,200 ± 900	6.5 ± 0.1
PLA-RP pellet	107,000 ± 3500	9.9 ± 0.4
PLA film	106,000 ± 4000	-
PLA-RP film	99,800 ± 4000	-

**Table 2:** Glass transition temperatures (Tg), cold crystallization temperatures (Tcc) and melting temperatures (Tm); enthalpies of crystallization (∆Hcc) and melting (∆Hm); and degree of crystallinity (Xc).

Sample	T_g_ (°C)	T_cc_ (°C)	T_m_ (°C)	ΔH_cc_ (J/g)	ΔH_m_ (J/g)	X_c_ (%)
PLA	58.5 ± 1.5 ^a^	120.4 ± 0.2 ^a^	150.4 ± 1.6 ^a^	29.6 ± 0.3 ^a^	29.7 ± 0.2 ^a^	0.11 ± 0.04 ^a^
PLA-RP	58.7 ± 2.0 ^a^	115.9 ± 0.4 ^b^	149.4 ± 1.8 ^a^	26.5 ± 0.5 ^b^	27.1 ± 1.4 ^a^	0.64 ± 0.06 ^b^

^a–b^ lowercase letters above indicate no significant difference between samples (*p* > 0.05).

**Table 3:** Static water contact angle, water vapor transmission rate and oxygen transmission rate.

Sample	WCA (°)	WVTR (g/m^2^·Day)	OTR.*e* (cm^3^·mm/m^2^·Day)
PLA	74.6 ± 1.4 ^a^	31.2 ± 1.1 ^a^	19.6 ± 1.3 ^a^
PLA-RP	74.7 ± 1.3 ^a^	32.7 ± 1.9 ^a^	22.3 ± 1.1 ^b^

^a–b^ lowercase letters above indicate no significant difference between samples (*p* > 0.05).

## Data Availability

The original contributions presented in this study are included in the article. Further inquiries can be directed to the corresponding authors.
